# Probiotic Supplementation During Human Pregnancy Affects the Gut Microbiota and Immune Status

**DOI:** 10.3389/fcimb.2019.00254

**Published:** 2019-07-16

**Authors:** Yuyi Chen, Zhe Li, Kian Deng Tye, Huijuan Luo, Xiaomei Tang, Yu Liao, Dongju Wang, Juan Zhou, Ping Yang, Yimi Li, Yingbing Su, Xiaomin Xiao

**Affiliations:** ^1^Department of Obstetrics and Gynecology, The First Affiliated Hospital of Jinan University, Guangzhou, China; ^2^Department of Obstetrics and Gynecology, The Third Affiliated Hospital of Sun Yat-Sen University, Guangzhou, China; ^3^Department of Pathology, The First Affiliated Hospital of Jinan University, Guangzhou, China; ^4^Department of Clinical Medicine, International School of Jinan University, Guangzhou, China

**Keywords:** pregnancy, probiotics, immunomodulation, machine learning, interaction network

## Abstract

The consumption of probiotics and fermented foods has been very popular in recent decades. The primary aim of our study was to evaluate the effect of probiotics on the gut microbiota and the changes in inflammatory cytokines after an average of 6.7 weeks of probiotic administration among normal pregnant women. Thirty-two healthy pregnant women at 32 weeks of gestation were recruited and divided into two groups. The probiotic group ingested combined probiotics until after birth. The base characteristics of the probiotics and control groups showed no significant differences. The structure of the fecal microbiota at the genus level varied during the third trimester, and administration of probiotics had no influence on the composition of the fecal microbiota however, many highly abundant taxa and core microbiota at the genus level changed in the probiotic group when compared to the control group. The analysis of cytokines showed that IL-5, IL-6, TNF-α, and GM-CSF had equal levels between the baseline and control groups but were significantly increased after probiotic administration (baseline = control < probiotics). Additionally, levels of IL-1β, IL-2, IL-12, and IFN-γ significantly increased among the three groups (baseline < control < probiotics). This result demonstrated that probiotics helped to shift the anti-inflammatory state to a pro-inflammatory state. The correlation analysis outcome suggested that the relationship between the microbiota and the cytokines was not strain-dependent. The gut microbiota varied during the third trimester. The probiotics demonstrated immunomodulation effects that helped to switch over to a pro-inflammatory immune state in the third trimester, which was important for labor.

## Introduction

Foods containing probiotics are all around us, and the use of foods containing probiotics in China is also increasing. Probiotics, defined as “living microorganisms, which when administered in adequate amounts, confer a health benefit on the host” (Hotel, 2001), may be especially useful to the human body. Among pregnant women, 1.3–3.6% use probiotics in the United States and Canada and up to 13.7% in the Netherlands (Rutten et al., 2016). Antibiotic treatment (Dethlefsen and Relman, 2011), dietary habits (De Filippo et al., 2010; Claesson et al., 2012; Matteo et al., 2012), aging and geography (Tanya et al., 2012) are all related to the diversity of the gut microbiota. Studies have shown that dysbiosis of the gut microbiota are related to the occurrence of diabetes mellitus, obesity, inflammatory bowel disease, and allergic diseases (Brown and Hazen, 2014; Logan et al., 2016; Sonnenburg and Bãckhed, 2016). Gestational diabetes mellitus is also associated with dysbiosis of the maternal and neonatal microbiota (Crusell et al., 2018; Wang et al., 2018). Probiotic supplementation and fecal transplantation are the most common methods used to modulate the gut microbiota and build a new balance in the microbiota community.

The immune system during pregnancy must maintain a tolerance to the fetal allograft and adapt to immune mechanisms against pathogens. The disruption of this balance will lead to miscarriage, preterm birth, preeclampsia, and other pregnancy-related complications (Bastek et al., 2011). During pregnancy, implantation and placentation of the embryo needs an inflammatory environment; then, the maternal body moves to an anti-inflammatory phase to help fetal growth and finally reverts to an inflammatory status that promotes delivery (Mor et al., 2017). A successful pregnancy depends on the ability of the maternal immune system to change and adapt to each specific developmental stage (Mor and Cardenas, 2010).

Decades of research has found that the key point of host-microbiota interactions depends on the immune system (Bunker et al., 2015; Longman and Littman, 2015; Honda and Dan, 2016; Blander et al., 2017). How the immune system changes during pregnancy (Aghaeepour et al., 2017) and whether probiotic supplements in pregnancy lead to a structural change of the intestinal microbiota and the responses of the host immune system are all unclear. The primary aim of our study was to evaluate the effect of probiotics on gut microbiota and inflammatory cytokines, which represent a change in immune functions after probiotic administration. The secondary aim was to observe the effect, if any, of probiotic administration during pregnancy.

## Materials and Methods

### Subject Recruitment

**Figure 1:**
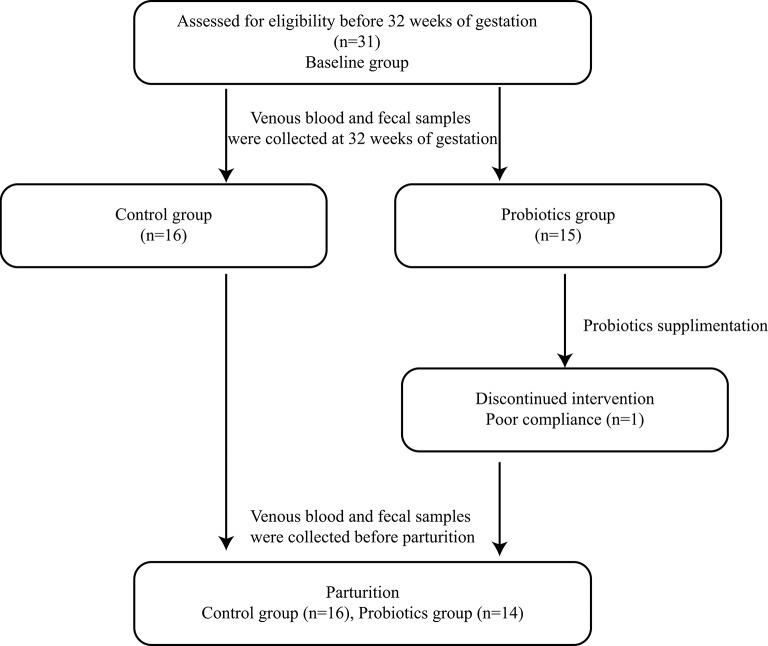
Flow diagram.

Pregnant women were recruited at The First Affiliated Hospital of Jinan University. Thirty-two normal first singleton pregnant women with no history of other diseases, especially periodontitis, type 2 diabetes and bacterial vaginosis, were recruited before 32 weeks of gestation and divided randomly into two groups; one participant was eliminated because of gestational diabetes mellitus detected in the third trimester, and another participant withdrew from the probiotic group before completion of the study because of poor compliance. Thus, 30 pregnant women finished the study: 14 pregnant women underwent probiotic administration, and the other 16 participants took no probiotics (Figure 1).

### Study Design and Sample Collection

The study project was authorized by the Institutional Review Board (IRB) for Human Subjects Research at The First Affiliated Hospital of Jinan University and the approval number was: 2019-011. All participants provided written informed consent prior to beginning the study.

Fourteen pregnant women were randomly assigned to the probiotic group, and the rest were assigned to the control group. After enrollment, pregnant women in the probiotic group received living combined *Bifidobacterium longum* (5 ^*^ 10^6^ CFU), *Lactobacillus delbrueckii bulgaricus* (5 ^*^ 10^5^ CFU) and *Streptococcus thermophilus* (5 ^*^ 10^5^ CFU) tablets produced by Neimengu Shuangqi Pharmaceutical Co., Ltd. Women in the probiotic group took two tablets twice a day (2 g/d) until delivery. Individuals in the control group took no pills. All participants were told that they were not allowed to take antibiotics or other foods contained probiotics during the experiment and to keep their doctors informed of any abnormalities. Participants were asked to return the packages of tablets to assess compliance. When any unused tablets were found in the packages, the participant were asked to adhere to the protocol; the total number of unused tablets was <10%.

Sampling operations were executed by trained professionals under strict aseptic conditions and a uniform protocol. Approximately 10 ml of peripheral venous blood and fecal samples were collected at enrollment. The first fecal sample was collected by the participants and stored in a household freezer (−20°C) until collected by our staff within 24 h. All participants underwent ultrasonography at term pregnancy to assess the fetus condition. When the pregnant women were admitted as labor began, additional peripheral venous blood and fecal samples were collected. All specimens were placed in sterile tubes, immediately frozen upon collection at −20°C, and then transported to the laboratory and stored at −80°C until used for total DNA extraction for later sequencing or testing.

### DNA Extraction and Sequencing

Total genomic DNA from fecal samples (~200 mg) was extracted using the CTAB/SDS method. DNA concentration and purity were monitored on 1% agarose gels. For each sample, we amplified the variable region four of the 16S rRNA gene using modified 515F/806R primers. All PCRs were carried out with Phusion® High-Fidelity PCR Master Mix (#M0531S, New England Biolabs, USA). The same volume of 1× loading buffer (containing SYBR green) was mixed with the PCR products, and electrophoresis was performed on a 2% agarose gel for detection. PCR products were mixed in equidensity ratios. Then, the PCR products were purified with the GeneJET Gel Extraction Kit (K0692, Thermo Scientific, USA). Sequencing libraries were generated using the Ion Plus Fragment Library Kit (4471252, Thermo Scientific, USA) following the manufacturer's protocols. The library quality was assessed on a Qubit 2.0 Fluorometer (Thermo Scientific). Finally, the library was sequenced on an Ion S5TM XL platform, and 400 bp single-end reads were generated.

### Microbiota Analysis

Single-end reads were truncated by cutting off the barcode and primer sequence, then the raw sequence data were filtered by Cutadapt (Martin, 2011) with a Phred score of <30. Vsearch (Rognes et al., 2016) (version 2.80) was used to remove the chimeric sequences and the replication of sequence data with repeat counts below eight times. Sequences were clustered into new operational taxonomic units (OTUs) by Usearch (Edgar, 2010) (version 10.0) at 97% identity, and representative sequences for each OTU were assigned a taxonomy based on the Greengenes 16S rRNA gene reference database. Sequences that failed to be assigned and singleton OTUs were removed. Alpha and beta diversities applied in the analyses were calculated by Usearch.

### Blood Chemistry

Serum was analyzed by the MAGPIX instrument (Luminex) using the ProcartaPlex Human Th1/Th2 Cytokine Panel (EPX110-10810-901, eBioscience, USA) and Serotonin ELISA Kit (ADI-900-175, ENZO, USA). The analysis included IL-1β, IL-2, IL-4, IL-5, IL-6, IL-12p70, IL-13, IL-18, interferon-gamma (IFN-γ), granulocyte-macrophage colony stimulating factor (GM-CSF), tumor necrosis factor-alpha (TNF-α), and serotonin. All analyses were carried out in duplicate following the manufacturer's instructions and analyzed by MILLIPLEX Analyst software. ELISA values were corrected for total cell protein in the plasma.

### Statistical Analysis

Basic information about the participants was assessed using Student's *t*-test, the Mann-Whitney test and the chi-squared test, depending on the type of data. An ANOVA or the Kruskal Wallis method with Bonferroni correction for multiple comparisons was fitted with alpha diversity and inflammatory cytokines between groups as appropriate. The correlation analysis between inflammatory cytokines and OTUs was calculated by Spearman's method, and the *p*-value was adjusted by the Benjamini and Hochberg method.

The taxa of the same OTU type were clustered at the phylum, class, order, family, and genus levels. The relative abundance of OTU > 0.1%, and more than 10% of participants were defined as having high abundant OTU. The different OTUs among groups were singled out by the edgeR (Robinson et al., 2010) package running on an R script, and a *p*-value, which was adjusted by the Benjamini-Hochberg method, less than 0.05 was considered statistically significant.

Core microbiota among groups were detected by the random forests machine learning algorithm (Breiman, 2001), which was “Random Forest” in the R package (version 4.6-14). The number of trees was set by the parameter “err.rate” to increase the accuracy, and the parameter “mty” was set to the default of 6.

## Results

### Characteristics of the Study Cohort

The baseline characteristics of the two groups, such as demographics, ultrasound parameters of the fetus, birth weight and 1-min Apgar score, showed no significant differences, as presented in Table 1. The duration of probiotics supplementation was 6.7 ± 1.6 weeks.

**Table 1:** Pregnant women and fetus characteristics.

**Demographics**	**Probiotic group** **(*n* = 14)**	**Control group** **(*n* = 16)**	***p*-value**
Age (year)	27.2 ± 3.2	27.8 ± 4.4	0.69
BMI at enrollment	26.4 ± 3.5	24.7 ± 2.2	0.12
Weight gained during the study (kg)	3.5 ± 1.5	2.9 ± 1.7	0.31
Gestational age at delivery (weeks)	39.6 ± 0.9	39.6 ± 1.1	0.94
Birth weight (kg)	3.3 ± 0.47	3.3 ± 0.37	0.98
Mode of delivery (%)			
Vaginal	11(73)	15 (94)	0.29
Caesarian section	4 (27)	1(6)	
Infant gender (male/female)	6/8	7/9	0.86
One-minute Apgar score (median)	9	9	
Ultrasound parameter of fetus			
Biparietal diameter (mm)	89.4 ± 3.2	94.3 ± 3.5	0.9
Head circumference (mm)	332.2 ± 8.4	327 ± 10.4	0.14
Abdomen circumference (mm)	335.9 ± 17.7	329.6 ± 17.7	0.32
Femur length (mm)	71.2 ± 2	70 ± 3.1	0.22
Index of umbilical artery resistance	0.52 ± 0.05	0.59 ± 0.24	0.30

### Fecal Microbiota Composition and Communication Analysis

**Figure 2:**
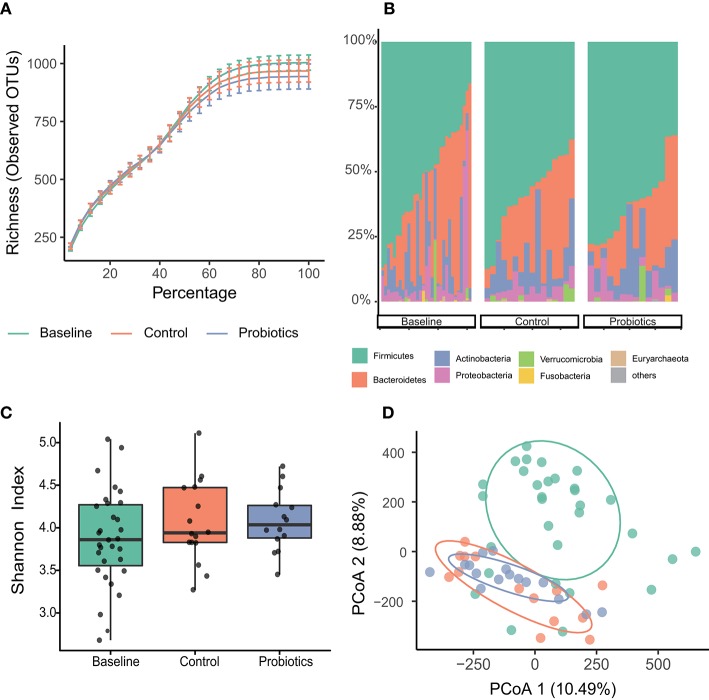
Microbial variations of fecal samples from the third trimester. **(A)** Species accumulation and rarefaction curves calculated for each group. **(B)** Relative abundance of bacterial phyla for each group. **(C)** Alpha-diversity estimated by Simpson indices showed no difference in groups. **(D)** Principal coordinates analysis (PCoA) based on Manhattan distances with ellipses representing 68% confidence intervals. Baseline vs. control: *p* < 0.001; baseline vs. probiotics: *p* < 0.001; control vs. probiotics: *p* = 0.464. Statistical significance was determined by the Adonis test (Vegan, R script package).

A total of 4,175,220 high-quality reads were found after filtration, and the median number of reads collected in the three groups was 67,647 (baseline, *n* = 31, range 41,879–84,258); 70,828 (control, *n* = 16, range 51,159–84,125); and 68,206 (probiotics, *n* = 14, range 49,984–86,195). The rarefaction curves showed that the sequencing depth was adequate, and sufficient OTUs were detected (Figure 2A). The high-quality reads belonged to 12,094 OTUs.

The top four abundant bacterial phyla in the fecal samples at baseline were Firmicutes (62.5%), Bacteroidetes (18.9%), Actinobacteria (11.9%), and Proteobacteria (5%). These bacteria added up to 98% of the sequences at the phyla level, and the other two groups shared the same tendency (Figure 2B). No significant differences were found among the three groups at the phylum level.

Measurements of within-sample diversity (alpha-diversity) were estimated by Simpson indices and showed no differences among the groups (Figure 2C). Beta diversity was assessed using unconstrained principal coordinate analyses (PCoA) of Manhattan distances to acquire interindividual differences in gut microbial communities. We found significant differences between the baseline and parturition groups in overall community structure (*p* < 0.001, in both groups); however, the control and probiotic group comparisons suggested that they were similar, and no significant changes were detected (Figure 2D).

### Supplementation With Probiotics Changed the Gut Microbiota

**Figure 3:**
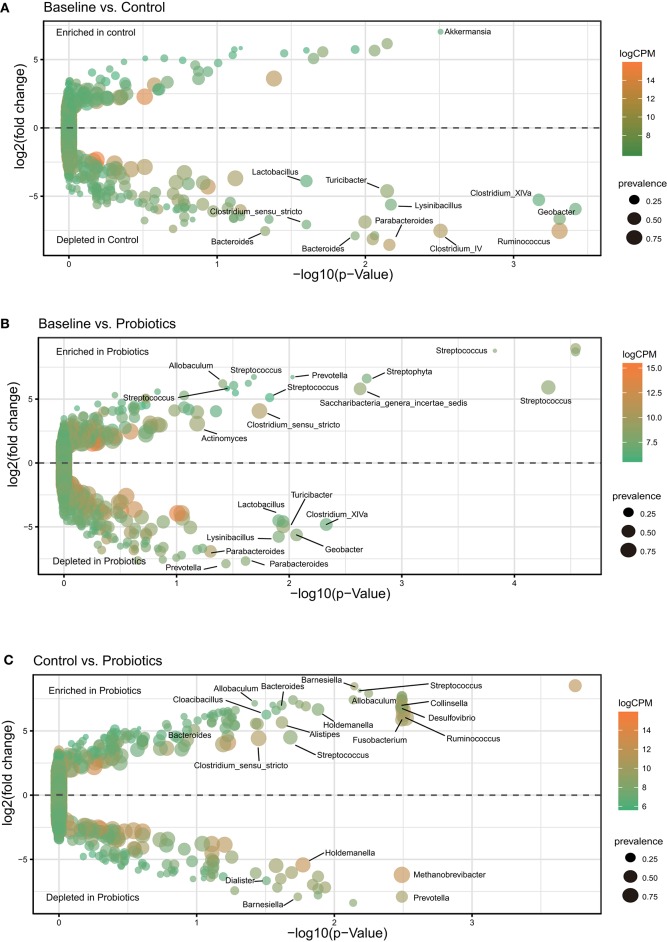
OTUs differentially abundant during pregnancy. Only the OTUs that were assigned to the Greengenes 16S rRNA gene reference database at the genus level and had significant differences were labeled. **(A–C)** Volcano plot of the OTU abundance demonstrates intragroup differences. The y-axis represents the log2-fold change of relative abundance calculated by EdgeR, and the x-axis is the *p*-value adjusted by the Benjamini-Hochberg method. CPM, counts per million; prevalence indicates the percentage of participants in which a given OTU is present.

Using a negative binomial generalized linear model by EdgeR analysis, we identified 68 OTUs changing from 32 weeks of gestation to antepartum, and six of them were highly abundant (Figure 3A). *Firmicutes* accounted for more than half of the OTUs (39/68) and the second most abundant bacteria was *Bacteroidetes* (25/68). Most of these bacteria (58/68) were depleted and varied during gestation with an average abundance from 0.03 to 0.0003%. Ten OTUs were enriched, and their mean abundance varied from 0.008 to 0.11%. After probiotic supplementation, analysis revealed that a total of 49 OTUs changed and five OTUs were highly abundant. Two-thirds of the OTUs (30/49) were depleted, with an average abundance from 0.02 to 0.0002%, and more than half were assigned to *Firmicutes* (18/30). Nineteen OTUs were enriched from 0.001 to 0.06% (Figure 3B). In the process of changing from 32 weeks of gestation to antepartum, the control and probiotics shared three OTUs, which belonged to *Streptococcus, Clostridium_sensu_stricto*, and *Ruminococcaceae*, and these bacteria were enriched in the probiotic group. There were 48 different OTUs between the control and probiotic groups, and 21 OTUs were highly abundant (Figure 3C). The top three depleted OTUs belonged to *Clostridiales, Clostridium_sensu_stricto*, and *Holdemanella*, and the most enriched OTUs were *Porphyromonadaceae, Holdemanella*, and *Lachnospiraceae*.

**Figure 4:**
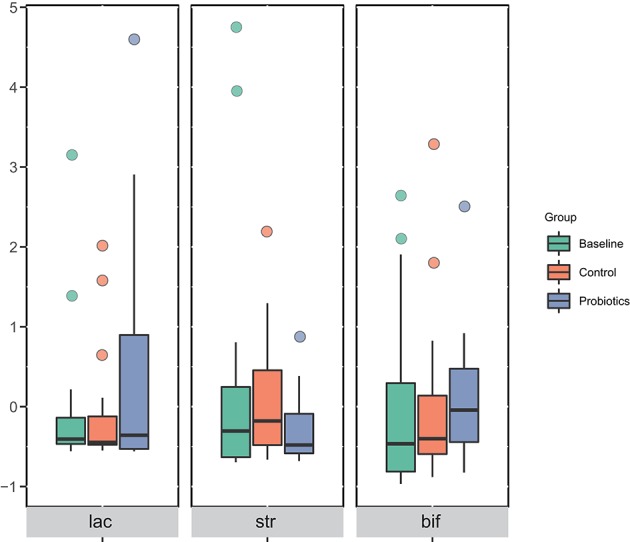
The corresponding bacteria of ingested probiotics varied in each group at the genus level. The data were processed for standardization using the Z-score method, and the y-axis represents the rate of change in relative abundance. lac, *Lactobacillus*; str, *Streptococcus thermophilus*; bif, *Bifidobacterium*.

Comparing to the control group, the relative abundance of *Bifidobacterium, Lactobacillus*, and *Streptococcus thermophilus*, which were the main ingredients of the probiotics, in the pregnant women in the probiotic group did not change. The corresponding bacteria showed no differences at the phylum, class, order, family, or genus levels among the groups (Figure 4).

### Core Microbiota Changed After Supplementation With Probiotics at the Genus Level

**Figure 5:**
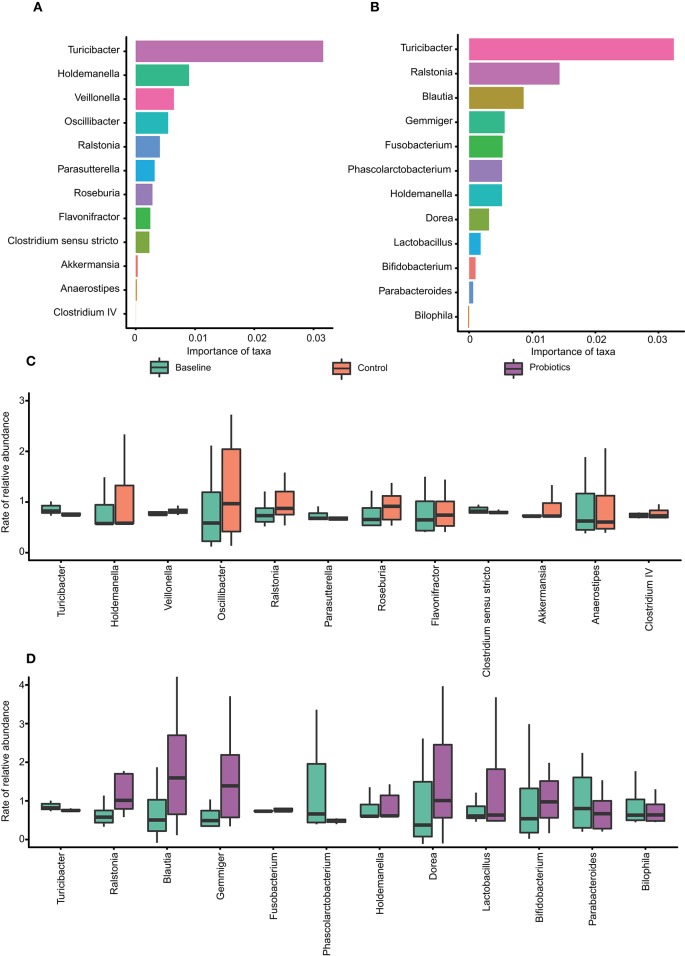
The core taxa selected by the random forest algorithm. **(A–B)** The feature importance scores of 12 taxa at the genus level, which can explain the variance among groups (baseline vs. control: 74.5%; baseline vs. probiotics: 80%). **(A)** Baseline vs. control; **(B)** baseline vs. probiotics. **(C–D)** The relative abundance of relevant taxa changes between groups. *p < 0.05. The data were processed for standardization using the Z-score method, and the y-axis represents the rate of change in relative abundance.

The relative abundances of the OTUs at the genus level were calculated by random forests classification to find the core microbiota in each group, and the models established by this machine learning algorithm could explain 74.5% of the microbiota variance between the baseline and control groups. Additionally, the variance among the baseline and probiotic groups explained by the algorithm was 80%. To reveal important bacteria at the genus level, 10-fold cross-validation was performed, and the error was the lowest when using 12 important taxa. The core taxa were selected by their feature importance scores, and the highest scores in both groups were for *Turicibacter*, with the other 11 being different (Figures 5A,B). In the control group, statistical analysis revealed that the relative abundance of *Turicibacter* and *Clostridium_sensu_stricto* were depleted and were significantly different. The same tendency was found in the probiotic group, in which the levels of *Turicibacter* and *Phascolarctobacterium* were significantly reduced (Figures 5C,D).

### Immune Responses Were Enhanced, and the Interaction Network Changed

**Figure 6:**
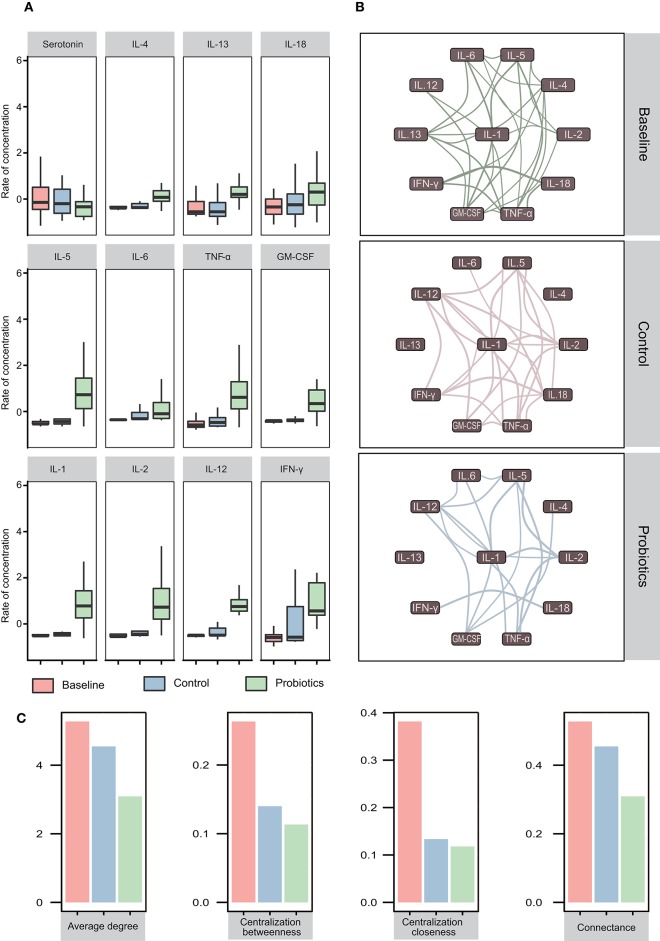
Cytokine variations and their interactions in pregnant women in the third trimester. **(A)** Twelve cytokines vary in blood samples. The first row showed no significant differences in the three groups. In the second row, the cytokine levels of the probiotic group increased, while the cytokine levels in the other two groups remained the same (baseline = control < probiotics). All cytokines increased, but the cytokines in the probiotic group increased more than those in the other groups (baseline < control < probiotics). *p < 0.05; **p < 0.01. The data were processed for standardization using the Z-score method, and the y-axis represents the rate of change in concentration. **(B)** Interaction network of cytokines in the three groups. The correlation value calculated by Spearman's method was >0.5, and *p* < 0.05 (adjusted by the Benjamini-Hochberg method) was retained. Edge width represents the correlation value. **(C)** The eigenvector index of the network demonstrates the variation tendency.

Eleven cytokines and serotonin were analyzed in this study, and we found that the levels of IL-4, IL-13, and IL-18 were not significantly different among the groups. However, for another four cytokines, IL5, IL-6, TNF-α, and GM-CSF, equal levels between the baseline and control groups were observed, which was followed by a significant increase after probiotic administration (baseline = control < probiotics). Additionally, the levels of IL-1β, IL-2, IL-12, and IFN-γ increased significantly among the three groups (baseline < control < probiotics) (Figure 6A).

To investigate the interactions and differences of cytokines in the three groups, we computed the correlations and drew the interaction network by igraph (R package, version 1.21) with a correlation cutoff of 0.5 and *p* < 0.05. The results revealed that all cytokines in the network were positively correlated and that the complexity of the cytokine network decreased from 32 weeks of gestation to antepartum. After probiotics supplementation, a less complex network was observed (Figure 6B). To quantify these differences, we calculated the eigenvector index of the network, and the number of edges among the baseline, control and probiotic groups were 29, 25, and 17, respectively. The indices of connectance, centralization betweenness and centralization closeness also had the same tendencies (Figure 6C).

### Association of Microbiota With Cytokines at the Genus Level

**Figure 7:**
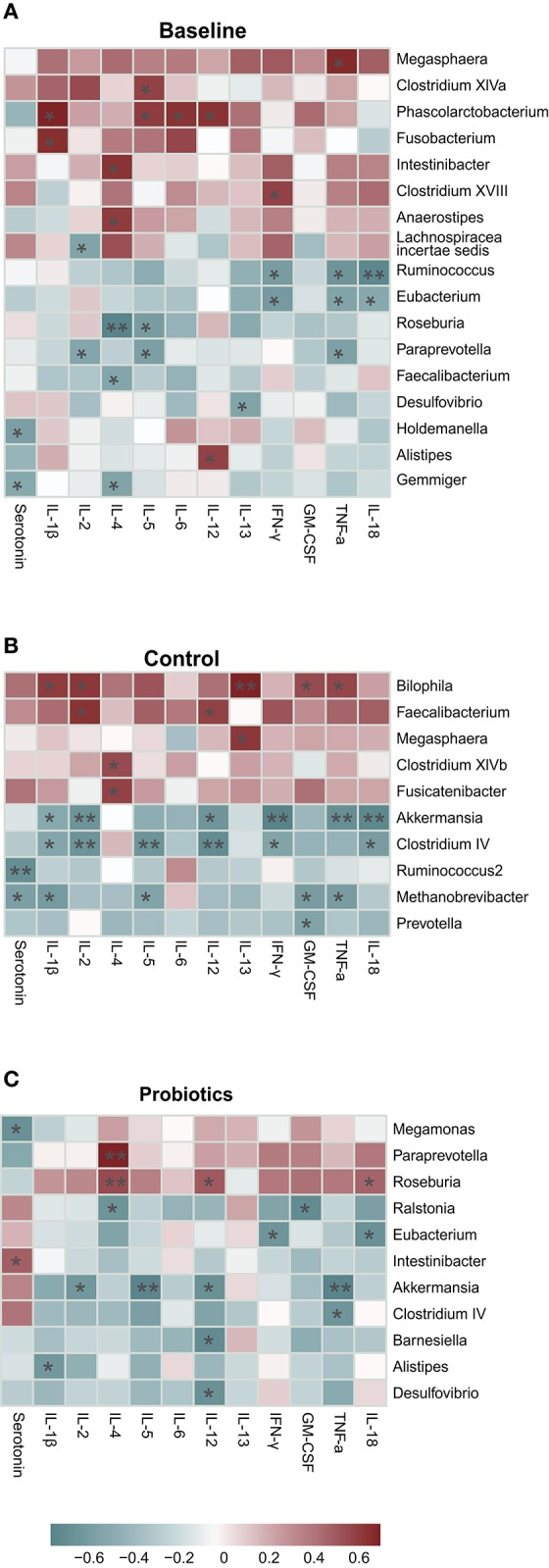
**(A–C)** High abundance taxa at the genus level correlated with cytokines (calculated by Spearman's method and *p*-values were adjusted by the Benjamini-Hochberg method. *p < 0.05; **p < 0.01). The average prevalence of groups: baseline: 97%, probiotics: 97%, control: 93%.

Spearman's correlation was used to identify the highly abundant taxa associated with the cytokines. In the baseline group, 17 taxa at the genus level were associated with cytokines, and half of them (8/17) were positively correlated. *Phascolarctobacterium* was positively associated with four types of cytokines, including IL-1β, IL4, IL-5, and IL-12. *Eubacterium* and *Ruminococcus* were found to be negatively associated with IFN-γ, TNF-α, and IL-18. It seemed that IL-4 was the most susceptible to changes in bacteria, and five taxa were associated with IL-4 (Figure 7A). At antepartum, only 10 taxa were related to cytokines, but the correlation index was greater and more significant than at the baseline. *Faecalibacterium* and *Megasphaera* were shared in the baseline and control groups, but the cytokines they related to were entirely different (Figure 7B). *Akkermansia* and *Clostridium*_*IV* had a strong negative relationship with six cytokines; however, *Bilophila* was positively associated with five cytokines. *Bilophila, Akkermansia*, and *Clostridium_IV* all affected the levels of IL-1β, IL-2, and TNF-α. After probiotic administration, we found 11 taxa, and most of them (8/11) were negatively associated with cytokines. *Akkermansia* and *Clostridium_IV* were found in the probiotic group, but their influence on cytokines was reduced and changed compared to the control group (Figure 7C). *Paraprevotella, Intestinibacter, Roseburia, Alistipes, Desulfovibrio*, and *Eubacterium* were found both in the baseline and probiotic groups, and their connection to cytokines completely changed, except *Eubacterium*, which was associated with IL-18 and IFN-γ.

## Discussion

Our study of probiotic supplementation in pregnant women showed no adverse clinical outcomes and that it was safe for fetuses, as determined in a previous study (Jarde et al., 2018). One study revealed that pregnant women with a high intake of probiotic milk products reduced the risk of spontaneous preterm delivery (Ronny et al., 2011). In contrast, a systematic review (Barrett et al., 2014) determined that relative risks of premature birth were elevated after probiotic administration. In our research, the gestation age between the two groups was equal, and no preterm birth was found in the probiotic group. The ultrasound parameters of the fetus at term pregnancy were not significantly different. However, considering the small sample size, adverse outcomes with a low incidence were hard to find in our study.

Fecal microbiota composition analysis revealed that most bacteria remained stable at the phylum level as gestation progressed. As shown in the PCoA, it was clear that the community structure of the bacteria changed from 32 weeks of gestation to antepartum, and probiotic supplementation had no influence on it. Supplementation with probiotics in adults has also been shown to not alter the composition of the microbiota (Singh et al., 2018). As the pregnancy progressed, the composition at the phylum level and the community structure of stool microbiota changed, and supplementation with probiotics did not interfere with these changes.

From 32 weeks of gestation to antepartum, the changed bacteria in the two groups were not the same, and most of the bacteria were depleted. Furthermore, the core taxa calculated by the random forests machine learning algorithm suggested that the features of the gut microbiota after probiotic administration had shifted. Contrary to our expectations, the relative abundance of *Bifidobacterium, Lactobacillus*, and *Streptococcus thermophilus* in pregnant women in the probiotic group did not change compared to the control group. Previous studies (Jafarnejad et al., 2016; Karamali et al., 2016) about probiotic supplementation in pregnancy also found the same results, in which probiotics had no influence on gut microbiota composition; however, we found in our research that some taxa at the OTU level, as well as the core microbiota, changed. We speculate that the complex interactions between bacteria are exerted through a systemic effect that may be the reason for these results. Although the relative abundance of probiotics remained stable, they did have an effect on the gut microbiota. There are three likely reasons for this observation. First, the stool samples were mainly from the lower gastrointestinal tract, and the probiotics ingested were distributed in the upper gastrointestinal tract. Probiotics usually enrich the upper gastrointestinal tract, and the components of bacteria in the lumen vary in different parts of the gastrointestinal tract (Zmora et al., 2018). Furthermore, *Bifidobacterium* and *Lactobacillus* are highly abundant in the gut microbiota, and the number of probiotics taken was too small; thus, the change in quantity was not noticeable. Finally, 16S rDNA sequencing can reliably identify bacteria only at the genus level and cannot distinguish strain-specific variations at the subspecies level.

**Figure 8:**
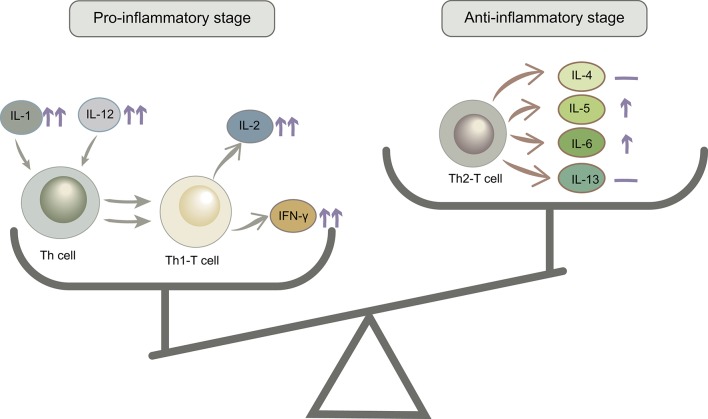
The anti-inflammatory/pro-inflammatory stage balance changed after probiotic administration. ↑↑, significantly increased; ↑, mildly increased; —, no change.

IL-1β, IL-2, IL-12, and IFN-γ levels increased from baseline to labor in the control group, and this trend was stronger in the probiotic group. IL-1β could stimulate T cell activation by upregulating the production of IL-2 and its receptor. IL-12 is a critical cytokine for T helper 1 (Th1) differentiation and induces proliferation and IFN-γ production by Th1 cells. IL-2 and IFN-γ are secreted by Th1 T cells, so increased levels of these cytokines could reflect the proliferation of Th1 T cells. The second trimester is characterized by an anti-inflammatory and T helper 2 (Th2) type immune microenvironment that is necessary for fetal growth, then the immune state will convert to a pro-inflammatory microenvironment that is important for labor and delivery (Mor et al., 2017). Additionally, IL-5, IL-6, TNF-α, and GM-CSF showed no differences in the control group but increased in the probiotic group. IL-5 and IL-6 are mainly produced by Th2 T cells, and TNF-α and GM-CSF are secreted by Th cells. However, IL-4 and IL13, which are produced primarily by Th2 T cells, remained stable after probiotic administration (Figure 8). According to the cytokine interaction network, after taking probiotics, the mutual relationship between the cytokines simplified as pregnancy advanced and became more distinct. These results revealed that probiotics could stimulate a variety of inflammatory cytokines and have immunomodulatory effects, which helped in the switch to a pro-inflammatory microenvironment in the third trimester, but we cannot confirm that this effect was beneficial to the pregnancy.

The multiple and complex interactions between cytokines and microbiota suggested that their relationship was not strain-dependent, and the correlation calculated by statistics could not explain their true relationships. Cytokines have multiple biological functions, including cell proliferation, differentiation and activation (Delves et al., 2017). Supplementation with probiotics did not change the microbiota structure, but the core bacteria that represented the features of gut microbiota shifted. This evidence indicated that probiotics altered some aspects of the gut microbiota that still need to be explored. The abundance of gut microbiota was easy to detect, but its interdependence, competition and effects on the host's immune system remain unclear. This observation may be the reason why the use of bacterial interventions to treat diseases have failed.

## Limitations

Our study had major limitations: first of all, the results may have limited generalizability based on the small sample size, which was the main limitation of the study. Secondly, because a placebo may have had potential side effects on the growing fetus, we set a blank control group instead of a placebo treatment group.

## Conclusion

The gut microbiota varies during the third trimester, and the structure of the gut microbiota remains stable after supplementation with probiotics. The probiotics have immunomodulatory effects that help to switch to a pro-inflammatory immune state in the third trimester. However, we do not know whether the pro-inflammatory state associated with probiotic supplementation is beneficial to the pregnancy. Prospective studies are warranted to explore the mechanism and its effects.

## Data Availability

The 16S rRNA sequencing data of the samples have been deposited in the China Nucleotide Sequence Archive (CNSA: https://db.cngb.org/cnsa) under accession code CNP0000301.

## Ethics Statement

The study project was authorized by the Institutional Review Board (IRB) for Human Subjects Research at The First Affiliated Hospital of Jinan University and the approval number was: 2019–011, and all participants provided written informed consent prior to beginning the study.

## Author Contributions

XX conceived the study. ZL, KT, HL, XT, YL, DW, JZ, PY, and YL collected the samples. YC and ZL conducted the experiments and analyzed the data. XX and YC prepared the manuscript. YS polished the manuscript. All authors read and approved the final version of the manuscript.

### Conflict of Interest Statement

The authors declare that the research was conducted in the absence of any commercial or financial relationships that could be construed as a potential conflict of interest.
